# Structured dataset of human-machine interactions enabling adaptive user interfaces

**DOI:** 10.1038/s41597-023-02741-8

**Published:** 2023-11-25

**Authors:** Angela Carrera-Rivera, Daniel Reguera-Bakhache, Felix Larrinaga, Ganix Lasa, Iñaki Garitano

**Affiliations:** 1 Faculty of Engineering, Electronics, and Computing. Mondragon Unibertsitatea, Arrasate-Mondragon, 20500 Spain; 2 Design Innovation Center (DBZ), Mondragon Unibertsitatea, Arrasate-Mondragon, 20500 Spain

**Keywords:** Technology, Engineering

## Abstract

This article introduces a dataset of human-machine interactions collected in a controlled and structured manner. The aim of this dataset is to provide insights into user behavior and support the development of adaptive Human-Machine Interfaces (HMIs). The dataset was generated using a custom-built application that leverages formally defined User Interfaces (UIs). The resulting interactions underwent processing and analysis to create a suitable dataset for professionals and data analysts interested in user interface adaptations. The data processing stage involved cleaning the data, ensuring its consistency and completeness. A data profiling analysis was conducted for checking the consistency of elements in the interaction sequences. Furthermore, for the benefit of researchers, the code used for data collection, data profiling, and usage notes on creating adaptive user interfaces are made available. These resources offer valuable support to those interested in exploring and utilizing the dataset for their research and development efforts in the field of human-machine interfaces.

## Background & Summary

Human-machine interfaces (HMIs) serve as platforms for cognition and communication between humans and machines. They play a crucial role in transmitting information^1^. The study of User Experience (UX) in this field aims to enhance usability, performance, and overall user satisfaction by considering user motivation goals during interactions. According to Aranburu *et al*.^2^, users are motivated by a sense of autonomy, competence, and their emotional connection to the system. To enhance the UX, manufacturing companies should consider factors related to user motivation, such as intuitive and efficient task completion and the system’s anticipation of user needs. The latter is closely tied to the cognitive capacity of the user, which translates into the amount of memory required for the user to meet their goals^3^. Manufacturers can take advantage of the analysis of user interactions through artificial intelligence techniques that can respond to user motivators.

This paper presents a dataset that includes sequences of human-machine interaction with several machine elements to perform a particular process. Sequential behavior can indicate the level of expertise or familiarity with a given device or machine. In the literature, multiple authors have studied user interactions to understand user tasks and goals in a wide range of fields. For instance, in engineering design, McComb *et al*.^4^ demonstrated how to assist designers with previous design iterations using sequence learning. On the other hand, methods to analyze human-machine interactions exploit machine learning techniques. Recommendation systems can provide adaptation and personalization for users, which can help reduce the cognitive load and increase the user’s positive feelings^3,5^. For instance, the use of deep recurrent neural networks and collaborative filtering algorithms can perform sequential recommendation of content and control elements^6^. Furthermore, the analysis of interaction sequences benefits not only end-users directly but also assists UI evaluators in providing suggestions to improve the evaluated interface^7^. However, despite the clear contributions of these studies, the datasets are typically unavailable to practitioners and scholars.

The dataset presented in this article was collected with the primary aim to enable the development of Adaptive User Interfaces (AUIs) that can deliver benefits across a range of industrial applications. AUIs are artifacts designed to dynamically adjust to operator interaction patterns^8^. This means that any repetitive set of actions performed by an operator to complete a task can be considered an interaction pattern that can be automated^9^. Leading to more personalized interfaces that enhance productivity^10^. Hence, improving usability and UX overall. The dataset can also be applied to a wider range of use cases, including sequence-aware and context-aware applications that utilize data generated by devices and users to provide customized experiences and self-adaptable designs. By leveraging multiple sources of context, such as task, time and user context, these applications can provide personalized interactions that increase user productivity^11^. Overall, this dataset has the potential to support the development of adaptive systems that better meet the needs of users.

## Methods

This section describes the data collection process. It starts by describing the design of the experiment and the setup, including a description of the acquisition and processing elements of the methodology.

### Experimental set up

The experiment was conducted using a machine in which multiple operators interacted through the same HMI to perform a mixture creation task. In this scenario, an industrial mixing machine from the food sector was utilized, which offers the advantage of being regularly used throughout the day by several users across two working shifts. Each time a mixture was ordered, the operator carried out a series of individual interactions with the HMI. These interactions were related to adjusting various parameters, including additive quantity, mixture type, and the use of containers. These parameters directly influenced the properties of the final product.

Users interacted with the machine through a mobile app that was specifically designed for the experiment. Operators accessed the app by scanning a QR code, after which they proceeded to select the required mixture. The captured interactions included two key components: **(i)** the order and sequence of steps the user followed, and **(ii)** the time interval in which the user interacted with the machine.

### Participants

Twenty-seven volunteer operators, aged between 23 and 45 years, participated in the experiment. Each operator granted formal consent to have their daily interactions recorded through the app. In total, 10,608 interactions were captured over a period of 151 days. All data was anonymized and does not contain sensitive user information.

### Data acquisition methodology

**Fig. 1:**
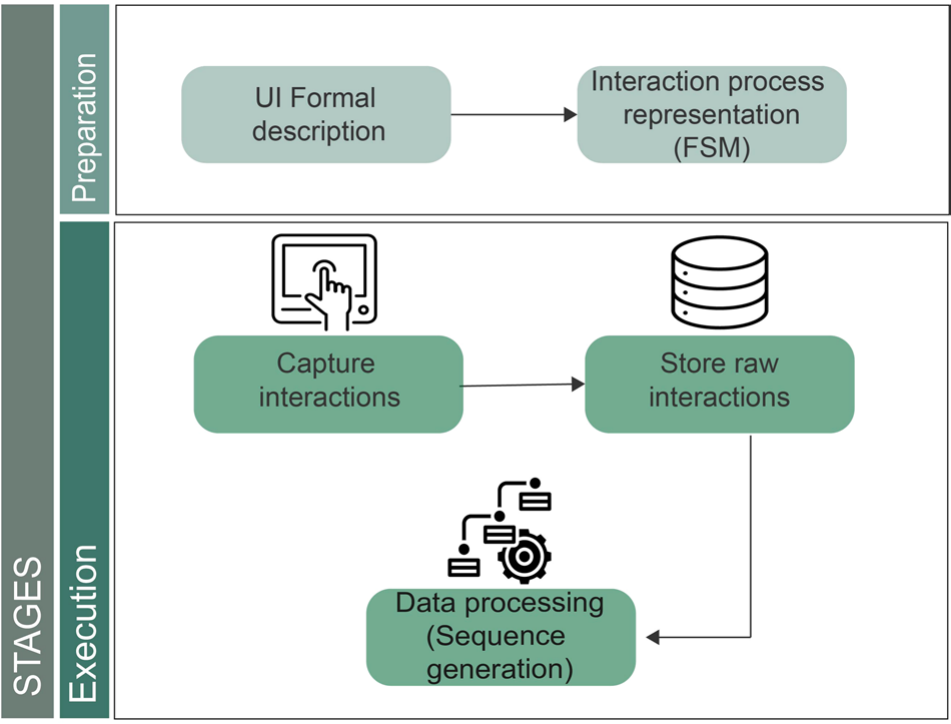
Data acquisition methodology.

**Fig. 2:**
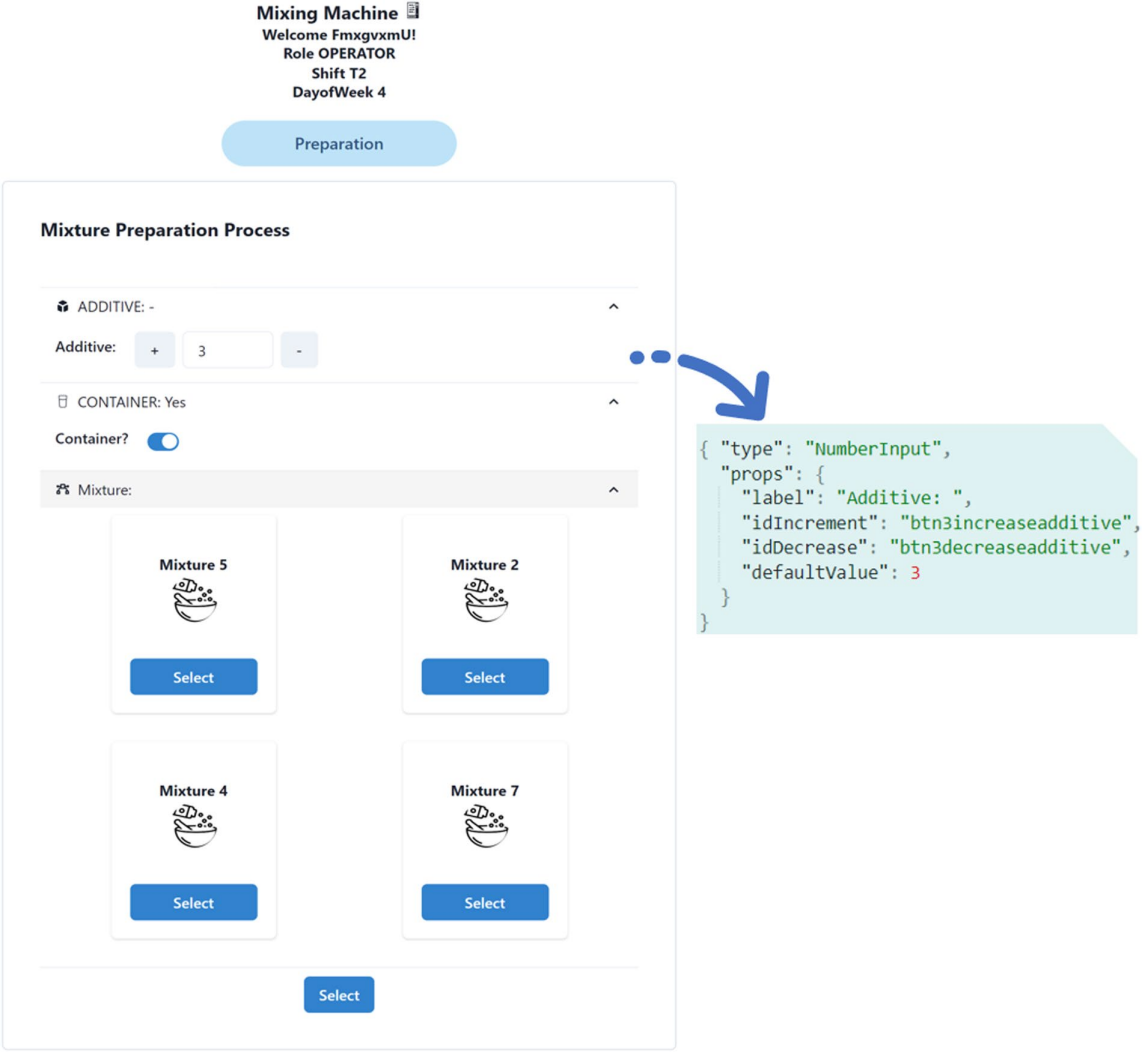
UIDL JSON description example of a UI.

Figure 1 illustrates the methodology for data acquisition, which begins with the *preparation* stage. This stage encompasses two steps: firstly, the user interface (UI) is formally described using a user interface description language (UIDL), which consists of a mark-up language that describes the entire HMI^12^. In this study, the JSON format was employed to represent each visual element in the HMI, with each element assigned a unique alphanumeric identifier.To provide an example of the UIDL utilized in this study, Fig. 2 displays a representation of the UI alongside its corresponding UIDL.

The HMI was implemented using Next.js, a React framework and Chakra UI. A dedicated function was created to programmatically generate the HMI using the user interface descriptor. The interface is designed to be responsive and can be used on tactile devices.

**Fig. 3:**
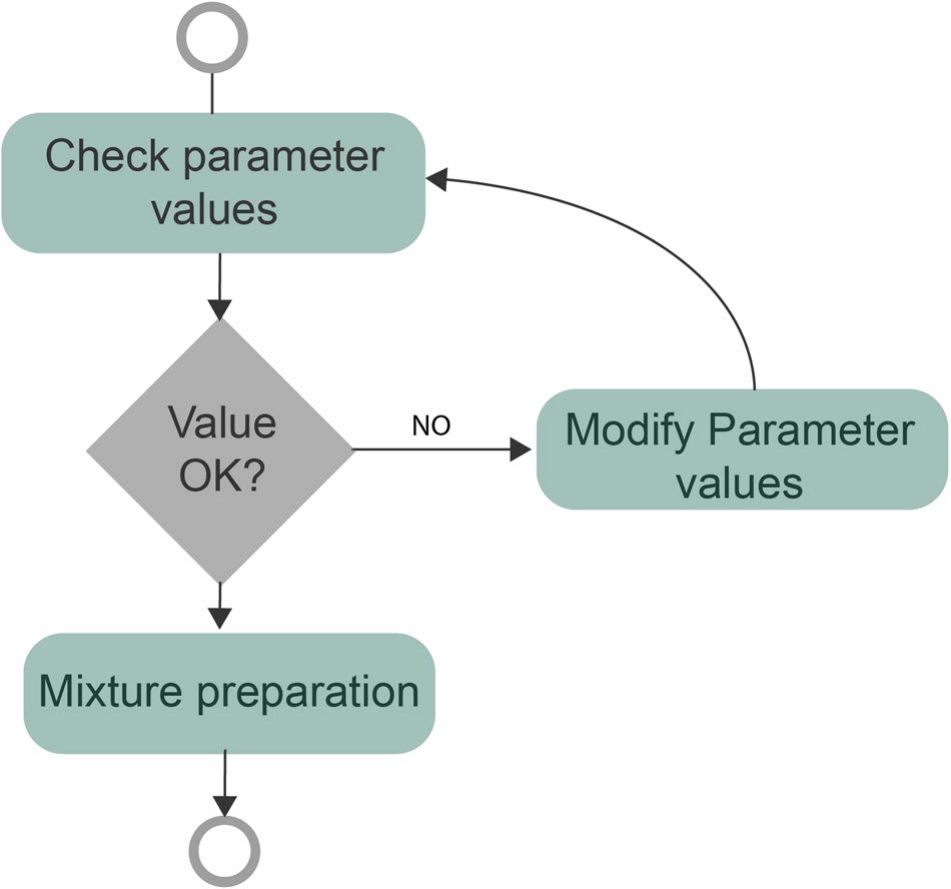
Interaction process representation (FSM).

Next, the *interaction process representation* required to prepare a mixture in the machine is described as a Finite State Machine (FSM), which is a model consisting of states, transitions, and inputs used to represent processes or systems. In this process, the user adjusts the parameters of a mixture until the values are considered correct (Fig. 3).

During the active phase of the experiment, when users access the machine using the application, a non-intrusive layer captures the interactions and stores them in a database (capture interactions). The information captured includes the user identity, the timestamp of the interaction in EPOCH format, and the identification of the interacted element (store raw interactions) (see Table 1). Once this information is collected, the data processing step generates the sequences.

**Table 1:** Example of raw operators interactions collected by the system.

SequentialID	UserId	Timestamp	ElementID
..	…	…	…
55	GCnLL0G8	1557994085106	BTN1OK
56	k09S54RA	1557994221803	BTN5Container
56	LbZs4Zb2	1557994221803	BTN5Container
57	k09S54RA	1557994230690	BTN1Additive
..	…	…	…

### Data processing

The goal of this step of the methodology is to generate valid sequences of interactions for each user. Perer & Wang^13^ define a sequence of events $E=\langle {e}_{1},{e}_{2},...,{e}_{m}\rangle$ (*e*_*i*_ ∈ *D*) as an ordered list of events *e*_*i*_, where D is a set of events known and the order is defined by *i*. This means that the event *e*_*i*_ occurs before the event *e*_*i*+1_. Additionally, in this process is considered that *E* must contain at least two events *e* to be accepted as a sequence^9^.

Using this definition and taking as input the raw interactions, it is possible to define valid interaction sequences as ${s}_{i}=\left[{e}_{begin},{e}_{1}^{i},\ldots ,{e}_{k}^{i},{e}_{end}\right]$ where *s*_*i*_ is a set of events and:

- The events *e*_*begin*_ and *e*_*end*_ are known, determining the beginning and the ending of the interaction sequence
- The variable *l* determines the length of the interaction sequence and its value should be > = 2

The sequences are extracted using the *“Valid sequences extractor”* algorithm presented by Reguera-Bakhache *et al*.^9^. As demonstrated in the FSM (Fig. 3), the interaction process initializes when an interaction occurs in any of the elements that allow the parametrization of the mixture and finalizes when the user clicks the button BTN1OK.

From the 10,608 interactions recorded, 1358 valid sequence interactions were generated. The composition of each interaction sequence is described in the following section.

## Data Records

The files can be downloaded from the **Figshare**^14^ data platform in a CSV format. The individual files are described below.

- **Raw interactions (raw_interactions.csv)**: Raw interactions represent each event performed by an user when interacting with an element of the machine, with their corresponding timestamp (see Table 1).
  - **Users(users.csv)**: This file presents information about the profile of users that participated in the study. Table 2 presents the distribution of the participants based on age, gender and role.

**Table 2:** Participants distribution.

Attribute	Item	Frequency %
Age	19–25	19%
	26–30	15%
	31–35	7%
	36–40	30%
	> 40	29%
Gender	Male	85%
	Female	15%
	Non-Specified	0%
Role	Supervisor	11%
	Operator	63%
	Senior Operator	26%

**Table 3:** User interactions sequences descriptor.

Type	Field	Description	Example values
User	user	Alphanumerical value that represents an user ID	FmxgvxmU
Context	shift	User work shift	
			t1 (Morning shift)
			t2 (Afternoon shift)
Time Context	initepoch	Timestamp when the interaction starts in EPOCH format	1557921970770
	endepoch	Timestamp when the interaction ends in EPOCH format	1557921987300
	initdayofweek	Day of the week of the interaction. Inferred from the timestamp. Integer value (0-6)	0 (Monday)
			1 (Tuesday)
	enddayofweek	Day of the week of the interaction. Inferred from the timestamp. Integer value (0-6)	0 (Monday)
			1 (Tuesday)
	isWorkingDay	is a work-day?. Boolean (1 or 0)	1 (work-day)
Metrics for Efficiency	timeOnTask	Time that a user needed to complete a task successfully in milliseconds. Integer value	8097
	numSteps	Number of steps performed by the user. Integer value	10
Task Context	mixture	Selected mixture on the machine. String value.	mixture1
	additive	Additive level. Integer value	0
	container	Use of container. String value.	Yes
	initialAdditive	Initial Additive. Integer value.	3
	machine	Machine ID. Alphanumerical value.	e11p3
Metrics for Effectiveness	anomaly	Error on task?. Binary value	1
User Interaction Sequence	interactionwu_prep	Sequence of user-initiated interactions with elements during a single mixture task	[‘btn1drink’, ‘btn5mixture3’, ‘btn5ok’, ‘btn1ok’]
	interactionwu_prep_json	*interactionwu_prep* in JSON format with a numeric ID that references SequentialID of *raw_interactions.csv* file	{775: ‘btn1drink’, 776: ‘btn5mixture3’, 777: ‘btn5ok’, 778: ‘btn1ok’}
- **User Interaction sequences (sequences_df_prep_EN.csv)**: Each row of this CSV file represents a valid interaction sequence to perform a task. This dataset was generated from the raw interactions following the “data processing” method previously described. Table 3 presents a description of the fields, classifying each field by the type of information they deliver, including some UX metrics relevant to the *efficiency* and *effectiveness* of the process. These represent more pragmatic aspects that focus on the task-oriented nature of an experience^15^.
- **User interface description (ui.json** & **hmi_elements.csv)**: The *ui.json* file serves as a JSON-formatted representation of every visual element within the study’s user interface. These elements are dynamically instantiated based on their component type. Table 4 provides a comprehensive overview of the various component types used, with the “interactive” column indicating which components are clickable for user interaction. To render the UI on the web application, we created a function in the app that reads and interprets the contents of the ui.json file. This allows to easily modify and update the UI as needed, without requiring significant changes to the underlying code (see sec:Code availability). The *hmi_elements.csv* is an informative file that lists the employed elements with their respective element IDs and component types.

**Table 4:** Type of components referenced on ui.json.

Element	Interactive	Example
VStack	N	A container that vertically arranges its children.
Heading	N	Typically used for displaying a title or heading, e.g., “Mixture Preparation Process”.
Accordion	N	A collapsible panel that contains multiple items, allowing users to expand or collapse sections.
AccordionItem	N	Each section or item within an accordion component.
AccordionButton	Y	A clickable button that expands or collapses an accordion section.
AccordionPanel	N	The content panel associated with an accordion item.
NumberInput	Y	An input field for entering numeric values that includes increase and decrease buttons, e.g., “Additive”.
YesNo	Y	A choice between “Yes” and “No” options.
SimpleGrid	N	A grid structure for arranging child elements in rows and columns.
Button	Y	A button that triggers an action when clicked, e.g., “OK”.
BasicCard	Y	A card container that is used to show and select available mixtures.

## Technical Validation

Sequential analysis can serve as an indicator of human expertise and behavior when performing both highly specialized tasks and common ones. To achieve this, the data must be consistent, complete, and preprocessed. For the latter, it was necessary to clean the data and ensure that its suitability for the intended use. This involved tasks such as removing duplicate interactions and correcting errors. Subsequently, starting from the raw interactions, the next step was to generate valid sequences, a process depicted in the subsection Data processing.

To validate the resulting dataset (*sequences_df_prep_EN.csv*) and facilitate the reuse, a data profiling analysis was conducted. First, it was important to guarantee the *consistency*, which could be defined as data presented in the same standard structure and its correctness in relationship with other data^16^. Therefore, it was validated that the elements within the interaction sequences correspond to the elements on the UI JSON file, and the users’ IDs exist in the *users.csv* file.

Other general aspects were evaluated using the IBM API of Data Quality for AI^17^, this toolkit offers a range of quality estimation and data profiling metrics to assess the quality of ingested data in an objective and systematic manner. These metrics produce a score between 0 and 1 that quantifies the presence of data issues, with a score of 1 indicating that no problems were detected. These metrics are designed for tabular datasets and accept input in the form of comma-separated values files^17^. The main used metrics are described in Table 5.

**Table 5:** Data Quality metrics score.

Metric	Description	Score
Data Completeness	Identifies missing values in the given data	0.99
Data Duplicates	Ratio of non-duplicate rows by the total number of rows	1
Data Homogeneity	Identifies format inconsistencies in non-numerical columns	1
Data Outliers	Identifies deviations in data from other observations.	0.98

**Fig. 4:**
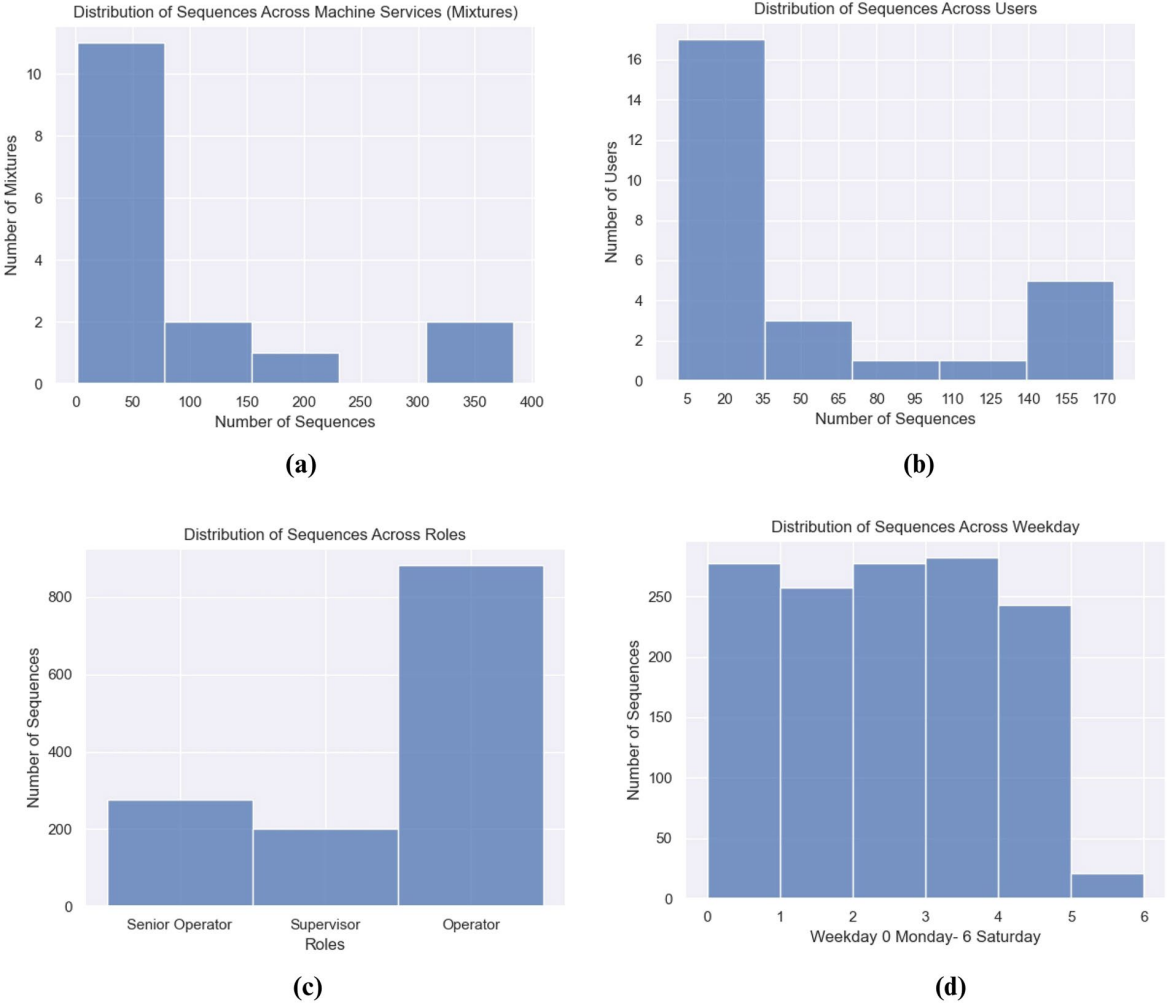
Sequences distribution (**a**) Distribution of Sequences Across Machine Services (**b**) Distribution of Sequences Across Users (**c**) Distribution of Sequences Across User Roles (**d**) Distribution of Sequences Across Weekdays.

The distribution of sequences was analyzed across different services, users, and time periods. Figure 4a, reflects that only a few services on the machine reach the maximum number of interactions, and the overall average of sequences per machine mixture is 84.85, whereas the median is 34.5. The data sparsity is an expected issue in applications that analyze user behavior, in which each user only interacts within a small set of items (i.e. recommendation systems)^18^. Similarly, in Fig. 4b there are users that engaged more with the application and record a higher number of interactions, having an average of 50.25 interaction sequences per user. These aspects must not be seen as a limitation in the utilization of the dataset but rather be a factor for data scientists to consider at the moment of developing and testing their models. Additional sources of contextual information regarding the interactions include the user roles and time periods. As the experiment was centered on machine usage, it was observed that the “operator” role accounted for the largest number of interactions (as illustrated in Fig. 4c), while weekdays exhibited a more evenly distributed pattern (Fig. 4d).

Density-based clustering algorithms can be used to determine the hourly distribution over time for operators. This is a type of unsupervised learning technique that identifies different clusters based on the density of the points. Each detected cluster in a 1-Dimensional space determines the time interval where the interaction is most frequent.

**Fig. 5:**
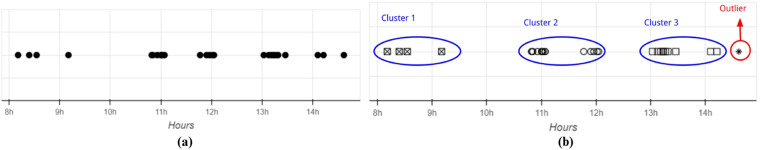
Distribution over time of interaction sequences (**a**) Distribution over time of interaction sequences from a single operator. (**b**) Identified clusters after applying Meanshift Algorithm for time interval detection.

To detect the clusters, first, we represented on a time axis each sequence from sequences_df_prep_EN.csv over time with a point. Second, MeanShift Algorithm^19^ is used to detect clusters and discard outliers. Figure 5a illustrates in a 1-Dimensional space the distribution of the interaction sequences from a single operator over time.

Figure 5b shows different clusters detected by Meanshift Algorithm for previous sequences. For each cluster, the leftmost point determines the beginning of the time interval, and the rightmost point the end of the interval. Each of these clusters represents the time intervals where the interactions are more frequent.

Overall, the analysis of the dataset can provide valuable insights into user behavior and usage patterns that can aid in the development of recommendation systems, adaptive user interfaces, or other applications. The insights obtained from analyzing the distribution of sequences across different services, users, and time periods can assist data scientists in the usage of the dataset to consider these factors.

## Usage Notes

### Generating Adaptive HMI

When designing AUIs, several key dimensions should be taken into consideration. Oestrich *et al*.^20^ presented a morphological box that structures these dimensions. In Table 6, the aspects are related to the data available on the dataset to highlight reusability.

**Table 6:** Mapping adaptive user interface dimensions to dataset fields.

Dimension	Options	Relevant Fields in Dataset
Goal of the Adaptation	Learning support	The dataset can enable the design of adaptive learning support systems by analyzing user behavior, interactions, and task completion times
	Performance support	The dataset can help in assessing user performance by analyzing the time taken to complete tasks, the number of steps performed, and the selected machine mixture. This information can be used to generate adaptations.
Adaptation Target	Presentation	Adjust the HMI based on interactions in the *interactionwu_prep* field. For instance, highlight frequently used elements or customize the layout.
	Instruction structure	Adapt the structure and order of instructions based on the observed patterns in the *interactionwu_prep* field. Ensure that instructions align with the user’s workflow. Another use case is the prediction of the parameters related to the task according to the *mixture*
Initiator of the adaptation	Specific user behaviour	Analyze the *timeOnTask* and *numsteps* fields to identify specific user behaviors, such as unusually task completion times. Identify by *role* or specific user
	Analysis of recent interactions	Analyze patterns in the *interactionwu_prep* field to detect trends in recent user interactions. These patterns can initiate relevant adaptations, such as offering assistance or altering the next steps.
Moment of Adaptation	Time-based	Use the *initepoch* and *endepoch* timestamps or *shift*. Implement adaptations based on time intervals derived from the timestamps.
	In between the current and next instruction	Analyze the *interactionwu_prep* field to identify breakpoints in user workflows. Insert instructions or adaptations at these points to guide users effectively.

However, defining the adaptation goal is key, whether it focuses on enhancing performance or providing instructional support, as it guides the entire process. The goal will define the techniques that can be used. From our previous works^10^, the analysis of the clickstream sequences (*interactionwu_prep* field) aimed to provide performance support by identifying recurring operator-machine interaction patterns and automatically detecting the time intervals during which these patterns manifest most frequently. This, in turn, enables the creation of “time-based” adaptations through the generation of Event Condition Action (ECA) rules. Additionally, these adaptations extend to altering the “presentation” of interface elements, specifically the layout order automatically. This is achieved through a Python-based engine that adjusts the user interface descriptor according to the rules. Notably, our research findings have demonstrated a reduction of over 40% in operator interaction time, showcasing the practical benefits of AUIs in enhancing efficiency and productivity. Facilitating the design process, we have employed a straightforward user interface descriptor in JSON format. This descriptor simplifies the modification process necessary for generating these adaptations, thus enhancing the usability and accessibility of AUI design. However, as indicated in Table 6, the adaptation target can vary, and the methods for presenting the adaptation can include overlaying cues or highlighting elements.

For instance, in order to provide learning support, one approach is to predict the next step based on past instructions and automatically execute these instructions. In such cases, the presentation of visual elements serves a communicative purpose. We have developed an example of next-step prediction using Markov Chains, which serves as a baseline approach for researchers interested in reusing this dataset. The associated code will be made available for use, facilitating further research and development in this area. However, the discussion of the results is out of the scope of this paper.

For further processing of the Human-Machine interactions dataset, we recommend well-known Python libraries such as Pandas and Scikit-learn.

## Data Availability

The code to *replicate the experiment* is available online. The frontend of the experiment was developed using Next.js and Chakra UI. Next.js is a React framework that enables server-side rendering, automatic code splitting, and other useful features for building web applications. Chakra UI is a component library that provides a set of customizable and accessible UI components to build user interfaces quickly and easily. The UI layout is described in the file *ui.json* and rendered on the app through the function *renderJSON*. The backend was implemented using Node.js and Express, with a PostgreSQL database used to store data. The frontend and backend communicate with each other through a RESTful API, providing a secure and efficient way for data to be transmitted between the two. Researchers interested in replicating the experiment can access the code and customize it to their needs, allowing for greater flexibility and control over the experimental setup. More information can be found in the Github repository (https://github.com/mu-sse/adaptiveUIs-project/tree/main/app-mixing-machine). The code for conducting *technical validation* has been developed using Python and is accessible through the GitHub repository provided in this document. To make use of the IBM API data quality for AI, a free sign-up process is necessary, which can be initiated at the following URL: https://www.ibm.com/account/reg/us-en/signup?formid=urx-50307. This sign-up process will provide access to the required API keys, as detailed in the accompanying documentation. For additional information and access to the code, please refer to the GitHub repository available online (https://gist.github.com/aicarrera/e6f99ea7f857de4c949afd2dfe1ff9be). Code *illustrating dataset usage* in the context of sequential recommendation (next-step prediction) through Markov chains has been developed using Python, building upon prior research^21^. More information is available online in the Github repository (https://github.com/mu-sse/adaptiveUIs-project/tree/main/HMI-sequence-recommender).
